# The benefit of quality control charts (QCC) for routine quantitative *BCR-ABL1* monitoring in chronic myeloid leukemia

**DOI:** 10.1371/journal.pone.0196326

**Published:** 2018-04-24

**Authors:** Birgit Spiess, Nicole Naumann, Norbert Galuschek, Sébastien Rinaldetti, Ute Kossak-Roth, Irina Tarnopolscaia, Elena Felde, Alice Fabarius, Wolf-Karsten Hofmann, Susanne Saußele, Wolfgang Seifarth

**Affiliations:** Department of Hematology and Oncology, University Hospital Mannheim, Heidelberg University, Mannheim, Germany; European Institute of Oncology, ITALY

## Abstract

Quantitative real-time polymerase chain reaction (qRT-PCR) is state of the art in molecular monitoring of minimal residual disease in chronic myeloid leukemia (CML). In this context, maintenance of assay fidelity and detection of technical inaccuracy are crucial. Beside multiple common negative controls for the clinical sample preparations, quality control charts (QCC) are a common validation tool to sustain high process quality by continuously recording of qRT-PCR control parameters. Here, we report on establishment and benefit of QCC in qRT-PCR-based CML diagnostics. The absolute quantification of *BCR-ABL1* fusion transcripts in patient samples is based on coamplification of a serially diluted reference plasmid (pME-2). For QCC establishment the measured Ct values of each pME-2 standard dilution (4–400,000) of a test set resembling 21 sequential qRT-PCR experiments were recorded and statistically evaluated. Test set data were used for determination of warning limits (mean +/- 2-fold standard deviation) and control (intervention) limits (mean +/- 3-fold standard deviation) to allow rapid detection of defined out-of-control situations which may require intervention. We have retrospectively analyzed QCC data of 282 sequential qRT-PCR experiments (564 reactions). Data evaluation using QCCs revealed three out-of-control situations that required intervention like experiment repeats, renewal of pME-2 standards, replacement of reagents or personnel re-training. In conclusion, with minimal more effort and hands-on time QCC rank among the best tools to grant high quality and reproducibility in CML routine molecular diagnosis.

## Introduction

The genetic key player of chronic myeloid leukemia (CML) is the reciprocal translocation between chromosomes 9 and 22 (t(9;22)(q34;q11)) resulting in a shortened chromosome 22 (Philadelphia chromosome). Depending on involvement of major, minor or micro breakpoint regions within the *BCR* gene, the majority of patients express different *BCR-ABL*1 mRNA fusion variants (most commonly e13-a2, e14-a2, e1-a2) resulting in the expression of fusion proteins with constitutively activated tyrosine kinase activity. The BCR-ABL1 fusion proteins affect various downstream signaling pathways and initiate reprogramming of the prior lineage commitment of hematopoietic stem and early progenitor cells [1, 2]. Furthermore, the BCR-ABL1 oncoprotein causes aberrant clonal hematopoiesis and triggers disease progression from chronic phase (CP) toward blast crisis (BC) [3].

Therapy regimen employing tyrosine kinase inhibitors (TKI) have considerably improved prognosis of CML [3]. Survival of responders is approaching that of the general population but lifelong TKI treatment is still recommended [4–6]. In several trials, TKI treatment has been stopped successfully in approximately half of the patients with deep molecular response. This has prompted the development of a new concept in the evaluation of CML patients known as ‘treatmentfree remission’ [6]. The depth of treatment response, i.e. the log reduction of detectable *BCR-ABL1* fusion transcripts, is an important factor in the decision to discontinue TKI treatment but depends on the definition of molecular response and the technical standardization of *BCR-ABL1* transcript measurements including selection of the best suitable control genes [7]. With current qRT-PCR techniques being able to reliably detect up to a 5-log reduction in *BCR-ABL1* using *ABL1* or *GUSB* as control genes, the CML Working Group of the ELN has recently proposed revised definitions of molecular response that take into account the sensitivity of the molecular test, that is, MR^4^ indicates ≥ 4-log reduction (BCR-ABL IS ≤ 0.01%), MR^4.5^ indicates ≥ 4.5-log reduction (BCR-ABL IS ≤ 0.0032%), and MR^5^ indicates ≥ 5-log reduction (BCR-ABL IS ≤ 0.001%), especially in negative qRT-PCR results. The prerequisite for valid calculation of major responses (log reduction) is a sufficient RNA quality as given by the absolute copy numbers of house-keeping gene *ABL1*. For MMR, MR^4^, MR^4.5^ and MR^5^, at least 10,000; 10,000; 32,000 and 100,000 *ABL1* copies are necessary, respectively [7, 8]. qRT-PCR (e.g. LightCycler (Roche) and TaqMan (ABI) technologies) meet all the requirements for sensitive and reliable diagnostic tools to perform molecular monitoring in CML patients [9, 10] and are routinously being used in our laboratory.

Therapeutic decisions within a longitudinal patient monitoring require the highest degree of qRT-PCR fidelity, accuracy, and reproducibility which must be checked by an adequate quality and validation management. As one of the German national reference laboratories for CML diagnostics we strictly work according the international guidelines for CML monitoring [11, 12]. Thus, it is to emphasize that separate controls are necessary for clinical sample preparation and qRT-PCR. A peripheral blood (PB) sample of a healthy person processed in parallel to patients’ samples served as negative control for each round of clinical sample preparation to avoid contaminants and false positive results. Beyond that, each qRT-PCR reaction must also be subjected to strict quality monitoring to verify that the *BCR-ABL1* quantitative assay is working within statistical control, while some degree of natural variability is unavoidable. Thus, a permanent monitoring of the experimental measurement precision should be able to signalize when the PCR assay is out of statistical control due to hardware failure, personnel bias, handling and pipetting errors, or to the use of a bad reagent.

Quality control charts (QCCs) according to Shewhart and Levey-Jennings are a suitable tool for implementing this requirement in analytical procedures [13–17]. QCCs graphically display whether a particular measurement is in control, i.e. control values fall between specific control limits, or is out of control.

Here, we report on establishment and benefit of QCCs in our CML diagnostics. The co-amplification of a serially diluted *BCR-ABL1/ABL1/GUSB* reference plasmid (pME-2) was monitored; Ct values were recorded for 564 PCR reactions and statistically evaluated leading to an adapted multilevel control system suitable for a consistent *BCR-ABL1* transcript monitoring. We demonstrate multiple advantages of using QCCs in CML routine quantitative *BCR-ABL1* monitoring e.g. as to decide when experiments should be rejected while at the same time avoiding false alarm situations. Furthermore, the best time point for renewal of reagents and the serial dilutions of standard plasmids as well as for the retraining of lab personnel can be determined by using QCC-based monitoring of the measurements.

## Materials and methods

### Clinical sample preparation and controls

As one of the German national reference laboratories for CML diagnostics we strictly work according the international guidelines for CML monitoring [11, 12]. For RNA extraction, the clinical samples were processed using the automated Maxwell^®^MDx technology (Promega, Mannheim, Germany). A peripheral blood (PB) sample of a healthy person processed in parallel to patients’ samples served as negative control for each processing cycle. This control serves for detection of cross contaminations and spill over during RNA extraction or cDNA synthesis. In case of false positivity the qRT-PCR data of all corresponding (processed in parallel) clinical samples was rejected and RNA was prepared again employing the frozen backup material. Control blood samples of healthy donors were obtained with written informed consent in accordance with the declaration of Helsinki from the local blood bank in fully anonymized manner. Since this study does not present patient data, an ethics approval is not required.

The serial diluted standard plasmid provided an external positive control for each LightCycler run. A PCR mix control served as negative control for each PCR experiment. For quality control of the sample material the internal reference transcript of *ABL1* of each sample was used. All controls were performed in duplicate.

### qRT-PCR on LightCycler platform

The LightCycler PCR and detection system (version 2, Roche Applied Science, Mannheim, Germany) was used for amplification and quantification of *ABL1* control and *BCR-ABL1* fusion genes. The PCR reactions were performed in glass capillaries employing a LightCycler “Fast Start DNA Master Hybridization Probes” kit (Roche Applied Science), *BCR* and *ABL1* specific primer and fluorescent probes as described by Emig et al., 1999 [9]. qRT-PCR for *BCR-ABL1* and *ABL1* transcripts was performed in duplicates using standardized cDNA of diagnostic specimens and of seven serial dilutions of 4, 10, 40, 400, 4000, 40,000 and 400,000 copies of standard plasmid pME-2 per reaction [18]. The limit of detection (95%) (LOD_95_) is 4 copies using serial dilutions of pME-2.

For a LightCycler experiment to be acceptable the machine generated data has to meet the passing criteria described in detail by Cross et al. [7]. The tolerable difference of the two single measurements of the standard and sample duplicates are Δ Ct < 0.5 in the range Ct ≤ 30, Δ Ct < 1.0 in the range of Ct ≤ 30.1–33 and Δ Ct < 1.5 in the range of Ct ≤ 33.1–37. Above Ct 37, there is no definition of a limiting maximum Δ Ct for the evaluation of duplicates [12]. If replicates show considerable variation and quantification may be unreliable, the respective experiment was reported as positive outside the quantitative range (POQR) and repeated.

### pME-2 standard plasmid containing *BCR-ABL1*, *ABL1* and *GUSB*

The pME-2 plasmid contains an 855 bp *BCR-ABL1* e14a2 / e13a2 derived PCR product (pBCR-ABL1^210^) as an insert in a pCR 2.1 TOPO Vector (Invitrogen, Carlsbad, CA, USA). The vector encodes also a 487 bp *GUSB* PCR product as an additional insert. The vector includes 3931 bp so the total pME-2 plasmid has a total length of 5273 bp [18]. The pCR 2.1 TOPO vector includes genes conferring ampicillin and kanamycin resistance.

### Plasmid preparation and dilution series

From a pME-2 glycerin stock solution stored at -80°C LBamp (Luria broth plus ampicillin) agar plate was inoculated and bacterial cultures were grown over night at 37°C. The final concentration of ampicillin in LB media was 50 μg/ml. Next day, a liquid overnight LBamp culture was inoculated with one bacterial clone and grown over night at 37°C. For the plasmid maxi preparation 250 ml LBamp culture were inoculated with 20 μl overnight culture and grown for 6–8 hours to a density of OD_595_ = 1.8. The chilled bacterial culture was harvested by centrifugation and plasmid DNA was prepared using the HiSpeed® plasmid maxi preparation kit (Qiagen, Hilden, Germany) according to manufacturer specifications.

After DNA preparation, the plasmid DNA concentration was determined using the NanoDrop (Thermo Fisher Scientific, Darmstadt, Germany) and 25 ng DNA were checked on a 1% agarose gel for the purity of the plasmid DNA. In case of pure plasmid DNA 5 μg of the DNA were linearized until completion using the restriction enzyme Not I (no 11014706001, Sigma-Aldrich/Merck). Adequate control PCR reactions and DNA sequence analysis (Sequiserve, Vaterstetten, Germany) were performed to ensure usability of the standard plasmid molecules. Finally a stock solution containing 2 x 10^8^ plasmid copies per μl was prepared and frozen in aliquots.

Originating from this stock solution the serial dilution series were prepared. For each diagnostic PCR reaction 2 μl of each serial dilution of the standard plasmid pME-2 equaling 4, 10, 40, 400, 4000, 40,000 and 400,000 copies per reaction were used.

### Analysis and evaluation of qRT-PCR derived QCC data

Ct values ("crossing points" (CP)) of the standard dilution series of the respective LightCycler experiments were exported into a Microsoft Excel sheet. These data was compared to the mean values and the respective warning and control (intervention) limits given by the respective QCC currently valid. Standard dilution data for each experiment must meet QCC requirements for passing. The analysis and documentation of the Ct values and the comparison to warning and control (intervention) limits is done automated by an algorithm implemented in our proprietary lab information management software (LIMS). Exceeding the limits is documented by our LIMS database (LeukoDB2). Fold changes based on Ct values were calculated according to Livak et al. [19].

### Statistics

Statistical calculations were performed using Microsoft Excel and GraphPad Prism according to standard procedures.

## Results

The sample capacity of LightCycler Version 2.0 resembles execution of 32 simultaneous qRT-PCR reactions each being performed in a separate capillary. In our setting, the first two capillaries contain the reaction master mix (without template) negative controls; seven pME-2 standard dilutions (2 x 10^0^−2 x 10^5^) in duplicates are measured in capillaries 3 to 16. Thus, there is space for eight patients to be analyzed in duplicates in capillaries 17 to 32. Within the investigation period presented here, 282 single PCR experiments were performed in total analyzing samples of 2256 patients. Of these, the first consecutive 21 qRT-PCR experiments served as training set for QCC establishment, the others are presented to demonstrate how quality assurance by application of QCC in daily routine works. Within the investigation period, five consecutive standard dilution series (A-E) were used each prepared from the same pME-2 stock solution. Only the *BCR-ABL1* qRT-PCR data obtained from measurements of the pME-2 dilution standards was evaluated for QCC establishment and quality assurance during routine diagnostics.

### Test dataset and control charts establishment

**Fig 1:**
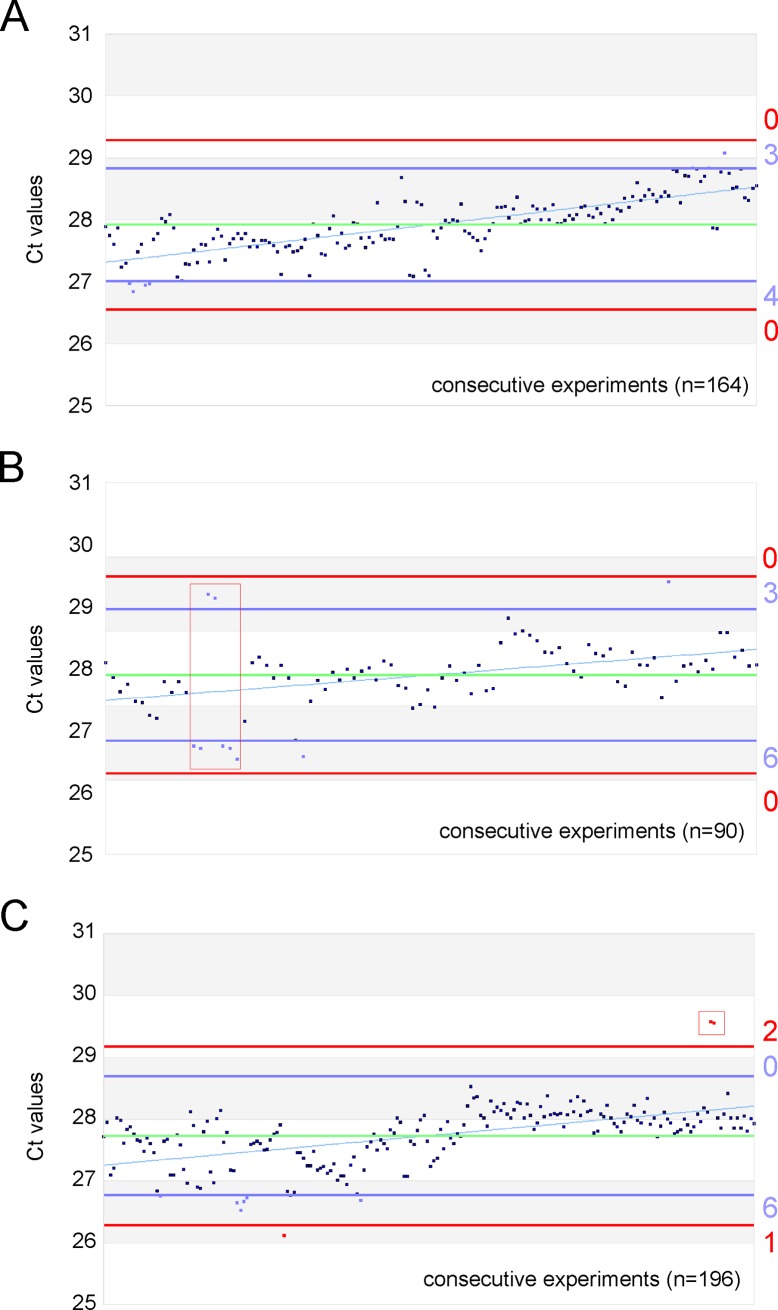
Quality control cards for the standard dilution 4000. The QCC feature the retrospective evaluation of Ct values for three workings solutions A, B and C including 164, 90, and 196 consecutive diagnostic PCR experiments, respectively. Green horizontal lines depict the mean, blue and red lines the warning (mean+/- 2SD) and control limits (mean +/- 3SD), respectively. Events during daily diagnostic routine that required special attention (warning limit violation in blue) or did not meet the pass/fail criteria (control limit violations in red) are shown as coloured data points. Data that led to repetition of entire experiments were boxed. The numbers of violations are given rightward.

For QCC establishment 21 diagnostic LightCycler experiments were performed providing the Ct values of the pME-2 standard series as training data set. As shown in Fig 1 the resulting values were graphically presented by plotting the respecting Ct values vs. the consecutive experimental numbers. Furthermore, warning and control limits and the criteria for passing were defined by calculating the mean and standard error of mean (SEM). A deviation of Ct +/- 2SD (SD = standard deviation) was defined as warning limit (blue lines). A deviation of Ct +/- 3SD was defined as control (intervention) limit (red lines) and requires an increased attention and monitoring by the operator as an out-of-control situation.

As out-of-control situations were defined in analogy to Westgard et al. [14]:

- Seven values in sequence increasing or decreasing.
- Seven values in sequence above or below the warning limit.
- Ten values in sequence hitting the warning limit.
- Two values in sequence outside of the control (intervention) limits (+/- 3SD).

Out-of-control situations require the following countermeasures:

- Repeating the run to exclude experimental errors.
- Repeating the run on an alternative device (if necessary, blocking and repairing the defective device).
- Using of new batches for all PCR reagents (including premixes, probes, and primers).
- When the situation persists: a new batch of the complete serial dilution series has to be prepared.

The QCC data set including the rules were implemented in our LIMS database system to enable an automated comparison with the standard series data of daily routine diagnostics. When the standard dilution Ct values meet the criteria of passing, then the patients diagnostic data is approved for the final findings report. Otherwise, a member of the scientific quality management (QM) team has to decide whether a run has to be repeated and what kind of counteractions have to be taken.

When analyzing the aspect of implementation of novel standard series in more detail, the dilution of fresh standard working solutions from stocks due to depletion make criteria for quality passing of a novel pME-2 standard series dilution necessary as well. The control of these criteria can also be performed by QCCs. The measured Ct values for the novel standard dilution series derived from a separate clearing experiment must be within the warning limits of the training data set (Ct +/- 2SD). Each dilution has its own training data set. Exemplarily, for standard dilution 4000 (i.e. 4000 copies of pME-2 per reaction) the acceptable Ct values are 28.02 +/- 0.42. During operation, the warning (Ct +/- 2SD) and control limits (Ct +/- 3SD) of each new standard series were recalculated and readjusted from the cumulative Ct values and the corresponding calculated standard deviations of the single dilutions of all past approved standard series.

### Application of QCCs during routine diagnostics

Of a total number of five consecutive standard working solutions (A-E) we graphically present three to discuss employment of QCC criteria in detail. Only the standard dilution 4000 is exemplarily shown in Fig 1A–1C. Each dot resembles one single PCR reaction performed as duplicate within the same experiment. Warning (Ct +/- 2SD) and control limits (Ct +/- 3SD) are indicated by blue and red horizontal lines, respectively. The mean is given by the green line.

The regression line (slope) indicates a decrease in PCR efficacy (i.e. increase in Ct values over time) due to the decay of pME-2 standard molecules within the working standard dilution 4000, a phenomenon characteristic for all working solutions.

The analysis of Fig 1A showed constantly increasing Ct values (n = 164 consecutive experiments) over time finally hitting the warning limit. According to the rules, when 10 values in sequence hit the warning limit, a new working solution of the complete standard series was prepared and the experiments were repeated.

Fig 1B depicts another event which did not meet the pass criteria of the QCC. Seven values in sequence were above or below the warning limit (n = 90 consecutive experiments). The respective data points are boxed in red. A in depth analysis found that pipetting inaccuracies were the cause of this high degree of scattering. Experiments were repeated and for future minimization of personal bias the lab staff was re-trained.

The evaluation of Fig 1C showed two peculiarities. On the one hand, the spread of the measured values (n = 196 consecutive experiments) changed after experiment no 106. On the other hand, two measured values of the same duplicate were in sequence outside the upper control limit (red boxed). The second event was an out-of-control situation and therefore led to the repetition of the concerning experiment and to renewal of the standard series. The reduction in the Ct value variance after experiment 106 was due to lab personnel rotation indicating that minimal personal bias can influence diagnostic performance and can be visualized by application of QCC.

### QCC determines timing of the standard dilution series renewal

**Fig 2:**
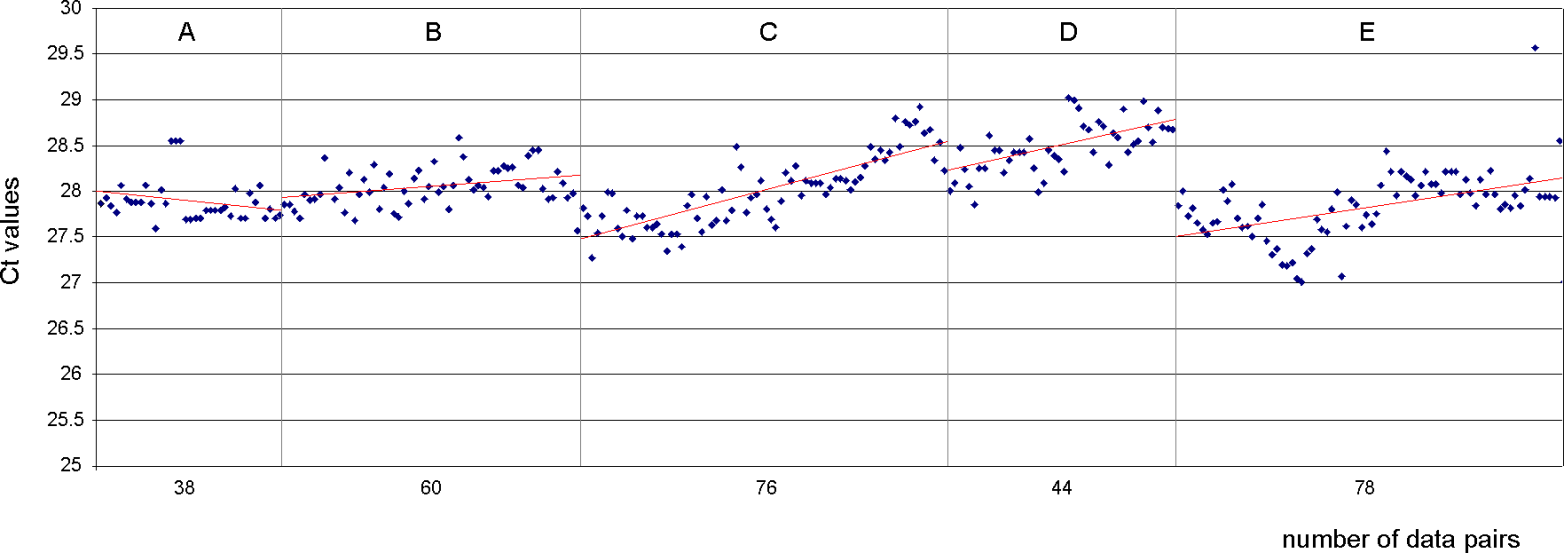
**Visualization of decay in the life span of five consecutive working solutions (A-E) of the pME-2 standard dilution 4000.** The Ct drift due to molecule degradation is shown by the increasing Ct values and is visualizes by the linear regression lines (red). For noise reduction and a more compact image size data point calculations were based on the mean of duplex PCR reactions.

As mentioned before, a decay of pME-2 standard molecules in all working solutions can be observed over time. In Fig 2, the decay of the standard dilution 4000 is shown for the periods of use of the five consecutive standard dilution series (A-E). The red regression lines indicate the decrease in PCR efficacy (Δ Ct/Δ t) due to the decay of the standard molecules in the working solutions. To analyze the mean decay of the standard dilution 4000 we determined the slopes (by calculation of the fold changes) based on the five consecutive dilution series resembling a total of 282 independent experiments (Table 1).

**Table 1:** Decay of plasmid working solutions.

A	B	C	D	E	
38	60	76	44	78	**working solution in use [d]**
0.137	-0.230	-1.035	-0.546	-0.68	**ΔCt**_**(init-end)**_
1.0996	0.8526	0.4880	0.6849	0.6252	fold change*
9.96	14.74	51.2	31.52	43.20	**total decay or increase [%]**
0.26	0.24	0.67	0.71	0.55	**decay or increase per experiment [%]**

Alterations of standard dilution 4000 and calculation of experimental decay based on 5 consecutive working solutions used for 282 independent experiments corresponding with the p210*BCR-ABL1* monitoring of 2256 chronic myeloid leukemia (CML) patients. Mean decay of working solutions used in phases B-E was 0.49% +/- 0.22 per one single experimental handling.

* calculation was performed according to Livak et al., 2001[19]

For calculation of the decay the average values of the measured duplicates for the standard dilutions were used. While for the period A no decay could be observed, the mean decay of working standard dilutions 4000 used in phases B-E was 0.49% +/- 0.22 under standard storage (4°C) per one single experimental handling. Similar decay rates were observed for all other standard dilutions underlining the importance of QCC for assessment of standard molecule integrity.

### Monitoring inter-experimental variance

**Fig 3:**
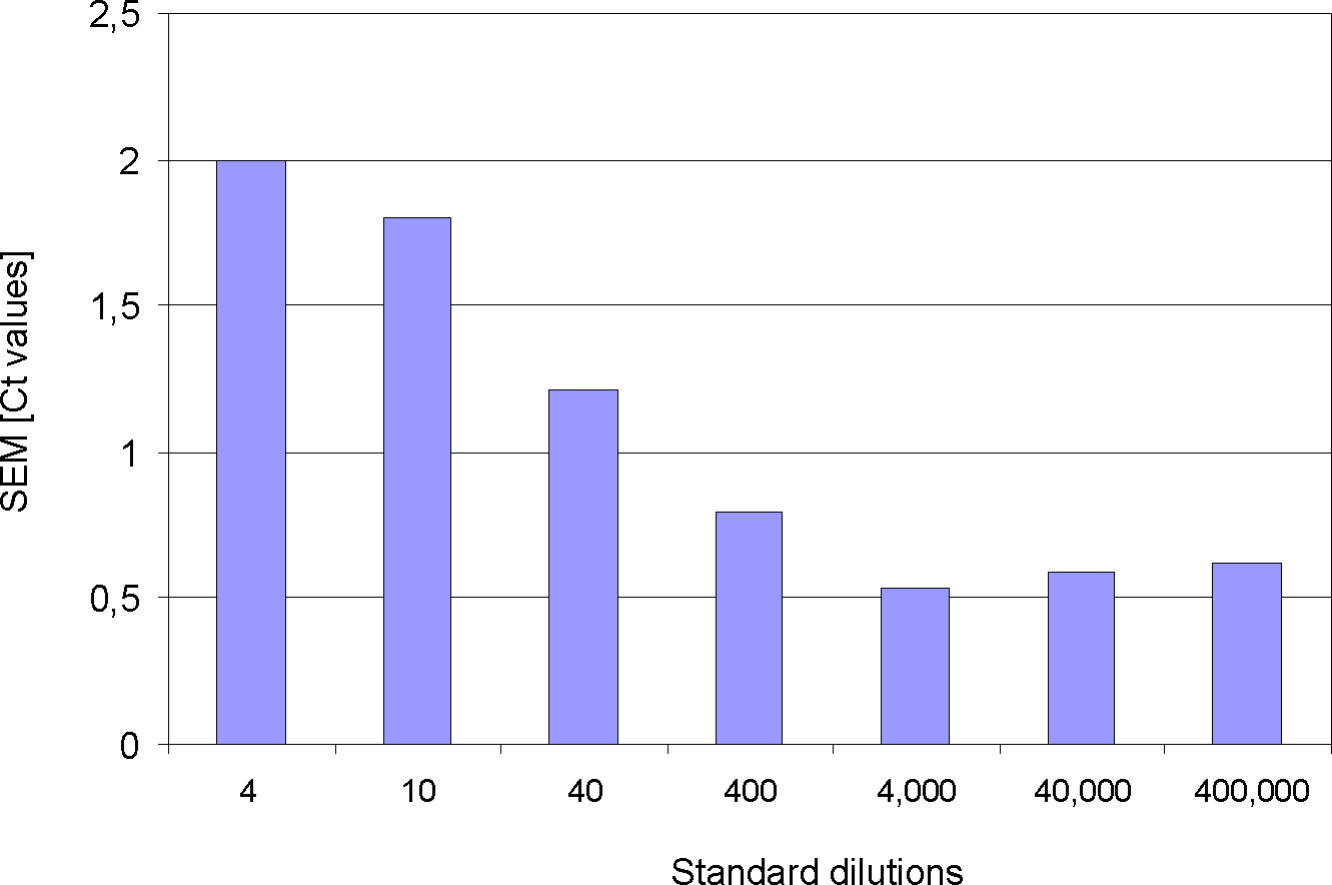
Inter-experimental variance for the seven pME-2 standard dilutions. Calculated were performed from 387 consecutive PCR experiments. Numbers on the x-axis correspond to the amounts of pME-2 plasmid molecules that serve as target within the respective PCR reactions. Decreasing numbers of target molecules inversely correlate with standard error of mean (SEM given in Ct values) due to pipetting inaccuracy.

The use of QCC in daily routine qRT-PCR are also suitable to assess the inter-experimental variance that can be an important parameter to monitor laboratory and machine precision. The Ct variance of all pME-2 standard dilutions from 387 individual PCR experiments was calculated and is depicted in Fig 3. When 4, 10 or 40 molecules have to be pipetted into a qRT-PCR reaction, the probability to withdraw the exact molecule number correlates with decreasing molecule concentrations. Therefore, the highest variance was observed in the standard dilution 4, where theoretically only 4 molecules were present in 2 μl of the working solution. Naturally, the smallest standard (4) displays the greatest variance. When pipetting such small amounts of molecules, even the smallest pipetting inaccuracies have a massive impact. Towards higher standard molecule concentrations the variance decreases continuously and reaches a plateau of about 0.5 Ct starting with standard dilution 4000.

## Discussion

We have established QCC in our diagnostic laboratory for routine quantitative *BCR-ABL1* monitoring in CML. Within the scope of international harmonization aspirations the intention of our manuscript is to focus on the benefit of QCCs within a functional diagnostic system established in 1999 [9]. Within this system, sample processing controls are routinely performed before carrying out the qRT-PCR experiments. For example, a peripheral blood (PB) sample of a healthy person processed in parallel to patients’ samples served as negative control for each processing cycle. The application of QCCs in addition to all necessary controls according to the international guidelines for CML monitoring [12] grants an additional benefit since it opens the possibility of a longitudinal monitoring of qRT-PCR performance. The implementation of QCC according to Levey-Jennings quality control methods combined with the introduction of a multilevel control system facilitates the stringent quality control of the diagnostic *BCR-ABL1* monitoring. Here, we present a practical field report that is meant to point out how a maximal benefit by investing minimal additional time and effort can be achieved by the use of QCC in a routine diagnostic setting where high assay fidelity (limit of detection), precision, and reproducibility of a PCR-based molecular assay are crucial as these quality parameters may influence therapy decisions.

In general, when presuming a Gaussian distribution for a number of identical consecutive experiments, 68% of the measured values are expected within one standard deviation (1SD) of the mean. 95% and 99.7% of all values should fall within plus or minus two (2SD) and three (3SD) standard deviations of the mean, respectively [14]. Therefore, it would be quite unlikely (0.3%) that one or even several consecutive measurements will fall outside 3SD of the mean. In this case, a careful method inspection including experiment repeat are required to identify systematic errors due to technical (machine) failure, personal bias or unperceived reagent decay.

Based on an initial training dataset we have established a multilevel control system, i.e. a set of rules, that–in analogy to Westgard rules (1977) [14]—helps discriminating between random and systematic errors, at the same time avoiding unnecessary experiment rejections, i.e. expensive and time consuming repeats of diagnostic experiments due to creation of false alarm situations. For the daily diagnostic routine, exemplified by the test set of 564 qRT-PCR reactions within the investigation period (A-E) presented here, the use of QCC is based on the recorded data of co-amplified serial dilutions of the pME-2 reference plasmid. The Ct values of any upcoming diagnostic qRT-PCR experiment are recorded and statistically evaluated with respect to the fixed QCC data and the rules leading to a consistent *BCR-ABL1* transcript monitoring.

Since the *BCR-ABL1* diagnostic system is based on absolute quantification adjusted to the plasmid standard dilutions, it is to emphasize that any bias in the standard curve will be reflected in the output data, i.e. the resulting *BCR-ABL1* transcript count in the patient sample. Thus, assuming that the pME-2 standard has decayed by half, the discrepancy between virtual (calculated) and the real plasmid concentrations will result in false (double) *BCR-ABL1* transcript counts in the clinical samples. In this case, patients will be diagnosed “worse” and this may contribute to future therapy options. A strict quality control is considered very important for monitoring of patients in treatment free remission status where the detection of MR^4^ and MR^4.5^ levels are indicative for therapy decisions. Thus, in our hands, the application of QCCs grants the best diagnostic performance for an optimized treatment of CML patients.

Therefore, it is of high importance to be able to verify during daily routine diagnostics that the plasmid standard dilutions are in best order and the qRT-PCR assay is working within statistical control accepting that some degree of natural variability between consecutive qRT-PCR reactions is unavoidable. Due to the possibility of recording longitudinal follow-up data (Ct values) QCC are the best tools to decide when experiments should be rejected while at the same time avoiding false alarm situations. Furthermore, the best time point for hardware inspection/repair, renewal of reagents and serial dilutions of standard plasmids as well as for the retraining of lab personnel can be determined by using QCC-based monitoring of the standard dilution measurements.

From our recorded data pool we have randomly chosen 5 data sets (A-E) each resembling the life span of one single standard dilution series to discuss some scenarios classified as out-of-control situations that led to intervention actions like repeating the experiments, renewal of the standard dilution series or personnel re-training.

The majority of pME-2 working solution preparations suffer from a distinct decay that contributes to 0.49% +/-0.22% loss of amplifiable pME-2 target molecules per single experimental handling. Despite storage and handling of the working dilution at temperatures at 4°C this decay seems unavoidable and may be caused by oxidation. Since the working solutions are used several times per day, frequent freeze and thaw cycles are contraindicated speeding up the decay, a well-known phenomenon [20]. To avoid working with progressively degrading pME-2 standards we set up as a QCC rule that when 10 Ct values in sequence are hitting or exceeding the warning limit, a fresh frozen aliquot of the standard working dilution should be used. These conditions are met between a number of 150 to 200 consecutive experiments making it reasonable to limit aliquot size to 450 μl (when using 2 μl of the standard dilution per qRT-PCR reaction). The standard dilution series A showed no increase of the Ct values over time. This phenomenon could have been due to the fact that the plasmid molecules within this working solution were not completely dissolved at the beginning of the analyses and became completely available during the progressing use of the standard series. In this case, the increasing solubilisation compensated for the oxidative decay leading to slightly decreasing Ct values. This had no impact on the diagnostic output data. All quality control error types described here, as identified by the use of QCC, enable us to monitor at any time assay fidelity (limit of detection) and precision of the diagnostic assay as the constant recording of the standard dilution-derived Ct values can serve as “vital sign” of the diagnostic system.

In conclusion, employment of QCC in our diagnostic lab has become a valuable tool for maintaining consistent quality control for the qRT-PCR based part of our CML routine clinical diagnostics during operation in daily business. With little more effort and hands-on time QCC open up the possibility of continuous monitoring of measurements and rapid intervention in case of out-of-control-situations. By implementation of QCC in our lab LIMS automated messages are being sent to the experimenter and the quality control team when during qRT-PCR data import the QCC rules are broken. By long-term Ct data recording systematic drifts potentially affecting the stability of the assay system can be detected with the option to counteract in time. Furthermore, the generation of printable monthly QCC reports can be used to meet the requirements of accreditation authorities. Thus, from our experience, we consider QCC as one of the best tools to grant a robust quality control of the qRT-PCR passing criteria in the clinical/diagnostic laboratory.
